# HIGHER STRESS AMONG ETHNIC MINORITY ADULT-CHILD CAREGIVERS COMPARED TO SPOUSAL CAREGIVERS IN DEMENTIA CARE

**DOI:** 10.1093/geroni/igad104.1387

**Published:** 2023-12-21

**Authors:** Jung-Ah Lee, Eunae Ju, Julie Kim, Eilleen Sabino-Laughlin, Seunghye Hong

**Affiliations:** University of California-Irvine, Irvine, California, United States; University of California, Irvine, Irvine, California, United States; University of California, Irvine, Irvine, California, United States; University of California, Irvine, Irvine, California, United States; University of Hawaii at Manoa, Thompson School of Social Work & Public Health, Manoa, Hawaii, United States

## Abstract

Ethnic minority family caregivers of persons with dementia (PWD) suffer from negative health conditions resulting from continuous care responsibility for PWD. While adult-child caregivers have additional family care and job responsibilities, cultural and linguistic needs in ethnic minority families further compound caregiving challenges. Little research examines ethnic minority caregivers’ health statuses. This study aimed to understand the emotional health status and dementia care experiences of minority caregivers, between spouses and adult-children. The study collected cross-sectional survey data on perceived stress and utilizations of caregiving resources: Alzheimer’s agencies, state-funded in-home supportive service, adult-day service, support groups, and caregiving education. Recruitment focused on diverse communities with special emphasis on minority caregivers who speak Spanish, Vietnamese, or Korean in California. Seventy-six caregivers participated in surveys in their respective language; 42% Korean, 38% Vietnamese, 20% Spanish; 49% spouses/partners vs 51% adult-children/grandchildren; 78% females; 55% PWD with Medicaid; Mean English Proficiency (EP) of 3/5(1.3). Sixty-four percent of adult-child caregivers were employed (full-time or part-time) compared to 5% of spousal caregivers (P<.05). Adult-child caregivers showed higher stress compared to spousal caregivers (P<.05) despite no differences in PWD’s activities of daily living (ADL) scores and instrumental ADL scores. Although EP of adult-child caregivers was higher than spousal caregivers (P<.05), both caregivers underutilized care resources and showed little awareness of Alzheimer’s agencies. Findings demonstrated higher caregiving stress of adult-child caregivers compared to spousal caregivers of PWD among minority groups. Tailored interventions with appropriate language and cultural aspects should consider the caregiver’s relationship to the PWD.

